# Annotations

**Published:** 1908-02-15

**Authors:** 


### ANNOTATIONS.

### The "Lancet" Libel Action.

In common with other representatives of the medical press, we offer our condolences with the editor of the *Lancet* upon the result of the recent action for libel which was brought against him in the King's Bench Division. The principle for which our contemporary fought in the case of "Tucker *v.* Wakley and Another" is one with which we are in entire agreement, and we much regret that an error in phraseology should have led to an adverse decision. We agree with a statement in the editorial column of the *Lancet* of February 1 that medical practice of the kind which was fully described in the evidence at the trial, by whatever term it is designated, must be condemned by the medical profession. We feel sure that the great majority of the members of the profession unite with us in expressing sympathy with the *Lancet*.

### Blood Pressure in Typhoid Fever.

It is refreshing to find that some, at least, of the familiar teachings of clinical medicine which have been handed down with engaging trustfulness throughout the centuries will survive the fierce light of modern laboratory methods. So many well-established bed-side observations have lately been contradicted by the latest instruments of precision that some physicians of the older school have begun to wonder whether the evidence of the unaided senses will be allowed to count for anything at all in the clinical diagnosis of the future. Recent investigations with the sphygmomanometer show that in typhoid fever the blood pressure is lower than normal, that it remains low until convalescence is established, and that it then returns to normal. Thus the sphygmomanometer endorses what has been taught for many years from the evidence of the fingers. This lowered blood pressure in typhoid fever, according to Barach, is due to a diminution in heart force and a reduction in vascular resistance, and not to the amount of fluid in the vessels. He further states that the blood pressure in typhoid bears no relation to temperature or pulse rate.

### Epileptic Colonies.

The problem of the regulation of the lives of epileptics is one that has long been before the medical profession. In the absence of any marked progress in our knowledge of the pathology of epilepsy, or in our treatment of it by drugs, it is important that we should keep constantly in mind a method of treatment which pays less attention to the frequency of the fits than to the social and intellectual condition of the patient. With few exceptions the epileptic is a man apart. Even when the fits are slight and do not forfeit the patient's occupation, the fact that he is an epileptic cannot be concealed and is usually a serious obstacle to success; while throughout his life he is never in complete harmony with his environment. In more marked cases these social disadvantages are much more prominent and distressing. It is, indeed, difficult to believe that the life of an epileptic under ordinary conditions can ever be a happy one. Even to his own family he is a perpetual nuisance, a perpetual source of anxiety—and he knows it. The method of dealing with epileptics by segregation, *i.e.* the "colony system," devised by Von Bodelschwingkh, is therefore, in the present imperfect state of our knowledge, at least a temporary and partial solution of the difficulty. Dr. Alan McDougall, writing in the *Medical Magazine*, draws attention to the advantages and limitations of this system as at present carried out. It seems quite clear that a certain proportion of epileptics are suitable for colony life, and that of these the great majority are happier and more useful under such conditions than they ever were before, while in about 10 per cent. there is a distinct and permanent improvement in the course of the disease.

### The Children's Bill.

On Monday last Mr. Herbert Samuel introduced into the House of Commons the new Children's Bill, which contains the Government's proposals for the consolidation, revision and amplification of the law relating to children. This Bill, which was read for the first time amidst cheers, is an attempt to extricate the various children's laws from their present state of confusion and inadequacy. To this end it is proposed to deal with the whole matter in one large and comprehensive measure, excluding only such controversial topics as education, licensing, and industrial employment. The Bill consolidates twenty-two statutes, and parts of many others, together with a number of new provisions. It is divided into five parts, which deal respectively with (1) Infant Life Protection—embodying and amending the Act of 1897, which was passed to diminish the evils of baby-farming and the perils of infants put out to nurse; (2) Prevention of Cruelty to Children—strengthening the present laws and facilitating the work of the societies, and constituting overlying an offence; (3) Juvenile Smoking—prohibiting the sale of cigarettes to those under the age of sixteen, suppressing smoking by them in public places, and authorising the confiscation of their tobacco; (4) Reformatories and Industrial Schools—consolidating the existing laws; and (5) the establishment of special children's courts for the trial of juvenile offenders, the enforcement of the attendance in court of parents, and the revision of the present methods of child-punishment, with especial reference to the unsuitability of common gaols. This important non-party Bill, of which we have given the barest outline, and to which we shall refer at greater length in a future issue, should command the sympathetic attention of all sections of the public. It should especially interest the medical profession, which has long sought for a thorough reform of the existing laws relating to the physical and moral welfare of children.

